# Risk of New-Onset Liver Injuries Due to COVID-19 in Preexisting Hepatic Conditions—Review of the Literature

**DOI:** 10.3390/medicina59010062

**Published:** 2022-12-28

**Authors:** Sandica Bucurica, Florentina Ionita Radu, Ana Bucurica, Calin Socol, Ioana Prodan, Ioana Tudor, Carmen Adella Sirbu, Florentina Cristina Plesa, Mariana Jinga

**Affiliations:** 1 Department of Gastroenterology, Carol Davila University of Medicine and Pharmacy, 020021 Bucharest, Romania; 2 Department of Gastroenterology, ‘Dr. Carol Davila’ Central Military Emergency University Hospital, 010242 Bucharest, Romania; 3 Department of Neurology, ‘Dr. Carol Davila’ Central Military Emergency University Hospital, 010242 Bucharest, Romania; 4 Centre for Cognitive Research in Neuropsychiatric Pathology (Neuropsy-Cog), Department of Neurology, Faculty of Medicine, “Victor Babeș” University of Medicine and Pharmacy, 300041 Timișoara, Romania; 5 Department of Preclinical Disciplines, Titu Maiorescu University of Medicine, 031593 Bucharest, Romania

**Keywords:** liver cirrhosis, SARS-CoV-2, COVID-19, hepatitis, cholangitis, chronic liver disease

## Abstract

The severe acute respiratory syndrome coronavirus 2 (SARS-CoV-2) impacted the world and caused the 2019 coronavirus disease (COVID-19) pandemic. The clinical manifestations of the virus can vary from patient to patient, depending on their respective immune system and comorbidities. SARS-CoV-2 can affect patients through two mechanisms: directly by targeting specific receptors or by systemic mechanisms. We reviewed data in the latest literature in order to discuss and determine the risk of new-onset liver injuries due to COVID-19 in preexisting hepatic conditions. The particular expression of angiotensin-converting enzyme 2 (ACE2) receptors is an additional risk factor for patients with liver disease. COVID-19 causes more severe forms in patients with non-alcoholic fatty liver disease (NAFLD), increases the risk of cirrhosis decompensation, and doubles the mortality for these patients. The coinfection SARS-CoV-2—viral hepatitis B or C might have different outcomes depending on the stage of the liver disease. Furthermore, the immunosuppressant treatment administered for COVID-19 might reactivate the hepatic virus. The high affinity of SARS-CoV-2 spike proteins for cholangiocytes results in a particular type of secondary sclerosing cholangitis. The impact of COVID-19 infection on chronic liver disease patients is significant, especially in cirrhosis, influencing the prognosis and outcome of these patients.

## 1. Introduction

Coronavirus disease 2019 (COVID-19), caused by infection with the severe acute respiratory syndrome coronavirus 2 (SARS-CoV-2), can affect people of any age and medical history, but it is known that patients with associated comorbidities have a higher risk of developing a more severe form of the disease [1].

The cumulative number of COVID-19 reached more than 604 million confirmed cases, with over 6.4 million deaths, according to published data in September 2022 [2].

SARS-CoV-2 infection may have different clinical manifestations, from asymptomatic to mild forms or severe multisystem involvement [3,4].

In 2021, it was estimated that approximately 1.5 billion patients worldwide suffered from chronic liver disease [5]. The World Health Organization (WHO) estimated in 2019 that 296 million people worldwide were living with hepatitis B and 58 million with hepatitis C, and that almost 1.5 million people for each type are newly infected every year [6,7]. The prevalence of non-alcoholic fatty liver disease (NAFLD) is constantly increasing, matching the rise in the prevalence of obesity [8].

Regarding liver dysfunction during COVID-19, although not all pathways are clear, it has been shown that up to 50% of patients have altered liver enzymes and hepatic impairment [4,9].

Pathophysiologically, SARS-CoV-2 has a direct cytopathic action, binding to the angiotensin-convertase 2 (ACE2) receptors, abundantly expressed in hepatic and biliary epithelial cells.

Histological studies found a variety of liver alterations: slight infiltration of lymphocytes in lobuli associated with a degree of dilation or thrombosis in centrilobular sinusoids, portal inflammation or calcification [10], and variable steatosis degrees going up to the fibrotic stage [11].

Vascular abnormalities and necrosis have been described in a number of studies, which are determined by venous endoluminal obstruction, lymphocytic endotheliosis, and hypoperfusion [12,13]. Fractions of the novel corona virus were identified in hepatic cytoplasm [14], and viral specific proteins were detected in cholangiocytes [15] and hepatocytes even half a year after infection [16].

The affinity of the SARS-CoV-2 virus for cholangiocytes and the biliary tract is high. The viral particles were also present in the gallbladder [17], and congestive alterations with a micro-thrombotic vascular pattern, congestion, and gallbladder mucosa friability were found, similar to SARS-CoV-2 hepatic damage [18].

From an immunological perspective, the relationship between the liver and SARS-CoV-2 is complex and combines the tissue direct viral action with the systemic pro-inflammatory response and “cytokine storm” [19].

ACE-2 receptors (ACE-2R), which are abundant in cholangiocytes and in the cells of the sinusoid endothelium and fewer in the hepatic cells [20], and trans-membrane protease serine 2 (TMPRSS2) are the two most important elements that contribute to SARS-CoV-2 infectiveness [21].

The virus can use an alternative pathway—furin, a protein convertase—to gain access to the host cells. The viral structure is composed of nucleocapsid (N), membrane, envelope, and spike proteins (S) [22]. As a result, the virion spike protein S can bind to the ACE 2 receptors and TMPRSS2 or use furin proteinase cleaving action.

The inflammatory and immune systemic response in SARS-CoV-2 infection is mediated by the breakdown of T cell lymphocytes, flare of interleukin 6 (IL-6), and tumor necrosis factor-α (TNF-α). The complement overstimulation pathway appears to be another mechanism that leads to hyperinflammation through the IFN-Janus kinase (JAK) 1/2-signal transducer and activator of transcription (STAT) signaling complex alongside nuclear factor kappa B (NF-kB) [23,24].

Acute liver injury (ALI) during systemic inflammation caused by COVID-19 corresponds to increased c-reactive protein (CRP) levels, elevated interleukin-6, and high ferritin [25]. Pro-thrombotic status induced by SARS-CoV-2 contributes to liver affliction through vessel endothelial injury, intravascular emboli, micro/macro thrombosis induced by immune dysregulation, platelet impairment, and hypoxemia [26].

Hepatic injury in COVID-19 patients can be assessed using liver biochemical tests: serum albumin, cholestasis markers-bilirubin, gamma-glutamyl transferase (GGT), alkaline phosphatase (ALP), liver transaminases-aspartate aminotransferase (AST/TGO), and alanine aminotransferase (ALT/TGP) [27,28]. A larger study found ALT and AST to be more than 3x the upper limit of normal (ULN) and bilirubin more than 2x ULN in patients with severe COVID-19, and AST to be higher than ALT, which can be a consequence of immune-mediated inflammation or injury [27,29].

The data presented by another study with more than 1000 patients showed AST serum values 3 times higher in patients with severe COVID-19 compared to those with the mild form of the disease [29,30]. It was observed that AST levels rise early in the disease course, and AST is more elevated than ALT [31,32].

One Chinese study published in 2021 evaluated serum albumin as a possible predictor for COVID-19 outcome [33]. Hypoalbuminemia was frequently found in infected patients, especially those with severe forms. Moreover, the level of albumin was correlated with the magnitude of the inflammatory status (lymphocyte, total number of T cells–CD4 and CD8, hemoglobin levels, and red blood cell numbers), but was not associated with CRP and AST [34]. Hypoalbuminemia is not a result of reduced synthesis in severe COVID-19, but a result of albumin consumption [35]. One study revealed that SARS-CoV-2 needs and utilizes albumin, so immediate albumin therapy is recommended for severe COVID-19 [36].

When it comes to the role of gamma-glutamyl transpeptidase (GGT) in COVID-19, it was found that increased GGT and AST are directly proportional to longer hospitalization. A study established an association between GGT and the expression of ACE2 connected to the common transcriptional hepatic nuclear factor-1β (HNF1B), which points out the possibility of using GGT as a biomarker for SARS-CoV-2 susceptibility [37]. A retrospective clinical study of 98 patients hospitalized at Wenzhou Central Hospital in Wenzhou, China, from January to February 2020, showed that 32.7% presented elevated GGT levels and 22.5% had increased CRP [38]. Moreover, a study that followed 118 COVID-19 patients for 376 days highlighted that elevated ALT, AST GGT, and body mass index (BMI) immediately after hospitalization were still increased 1 year after discharge, which emphasizes the need for close monitoring of patients with liver abnormalities [39].

## 2. Materials and Methods

The main purpose of this review was to establish, from the latest published data, the impact of COVID-19 infection on preexisting liver conditions.

We searched the databases PubMed, National Library of Medicine, the World Health Organization International Clinical Trials Registry Platform (ICTRP), the World Health Organization International Clinical Trials Registry Platform (ICTRP), Google Scholar, and Google during the pandemic era, starting from the beginning of 2020, until now, September 2022, for articles written, especially, in English that described the effects of COVID-19 infection on preexisting liver conditions. The search was carried out using the search terms “coronavirus”, “COVID-19”, and “SARS-CoV-2”, combined with “liver”, “hepatic”, “cirrhosis”, “NAFLD”, “hepatitis”, “liver tests”, “cholangitis”, and “inflammation”.

Research articles, reviews, case series, and case reports were taken into consideration, and almost all of the 123 articles cited were analyzed further. We excluded articles that included hepatocellular carcinoma, as any alteration in this stage can influence the course of the disease. Drug-induced liver injury (DILI) articles were excluded. The multitude of DILI can be a separate article because of the large number of medications used to treat COVID-19 infection with possible hepatic alterations that may overlap with the drugs used in preexisting liver diseases.

We paid attention to articles connected to liver injuries provoked only by COVID-19 but with no further evaluation.

The secondary sclerosing cholangitis determined by COVID-19 was included even though it is related to the cholestatic effect of COVID-19, and only case series and case reports were found.

## 3. COVID-19 and Cirrhosis

Cirrhosis is the final stage of long-term liver disease, causing an alteration in liver architecture through processes such as the production of extensive nodules, neo-angiogenesis, vascular restructuring, and newly formed extracellular matrix deposits [40]. Chronic liver disease is characterized by a cyclic process consisting of inflammation leading to the destruction and subsequent regeneration of liver parenchyma [40]. Cirrhosis may develop as a consequence of this process over the years, but at the time the diagnosis is made, it is considered irreversible [40]. Chronic liver disease causes immune dysregulation and inflammation, which can have an augmented effect on the processes present in SARS-CoV-2 infection [41].

The expression of ACE2 in hepatocytes of cirrhotic patients is 30 times higher than in the normal liver, and the association of metabolic syndrome increases the expression of both ACE2 and TMPRSS2 [42].

COVID-19 might affect the liver directly [43] (hepatocyte destruction caused directly by the SARS-CoV-2 virus or through viral translocation from the gut to the liver) or by indirect mechanisms [43] (such as systemic inflammation, hypoxemic effects on preexisting liver diseases, or ICU admission length) [43].

SARS-CoV-2 infection and cirrhosis seem to be a fatal combination, with augmented immune dysregulation being at the core of further biological processes leading to a more severe form of the disease [41]. Inflammation, in this case, is predominantly initiated by circulating active immune cells and pro-inflammatory cytokines [44].

One study estimated the overall mortality in patients with cirrhosis and SARS-CoV-2 association at about 32%, the mortality and morbidity increasing for patients with a higher Child-Pugh score, a widely used tool to assess the prognosis of patients with cirrhosis [44]. In this study, Child-Pugh class C patients had a 21% survival chance if admitted to the intensive care unit (ICU), dropping to 10% if they were on mechanical ventilation [45]. Another study revealed that mortality is also influenced by different risk factors such as advanced age, obesity, type II diabetes (T2D) or cardiovascular diseases; 78.7% of COVID-19 patients had lung-related conditions as the cause of death, while 4.3% of deaths were caused by cardiovascular disease and 12.2% by liver conditions [45].

The results from the COVID-Hep registry data showed that patients with fibrotic stage liver disease have a higher death rate [44]. This analysis included 745 patients, 386 with cirrhosis, showing a 32% mortality in this group, which is 4 times higher compared with non-cirrhotic ones, with an overall mortality of 20% (150 deaths/745 patients) [44]. The risk factors were higher Child-Pugh scores (C vs. A—51% vs. 19%), age, being Caucasian (OR 2.52; 95% CI 1.73–3.68; *p* < 0.001), cardiovascular comorbidities, and renal function impairment (OR 1.19 per mg/dl; 95% CI 1.04–1.38; *p* = 0.014) [44].

Moreover, another study confirmed that the Child-Pugh class can be used to assess the prognosis of SARS-CoV-2 infection by determining the risk of decompensation—64% for the C class compared to 30% for the Child-Pugh A class [46]. It presented as ascites or aggravation of the current ascites in 109 patients (28%), portal encephalopathy, rupture of varices in 27% of cases, and 3% with spontaneous bacterial peritonitis [46].

The death rates for patients with fibrotic stage liver disease were evaluated in large studies worldwide, and a mortality between 25 and 51% was found for cirrhotic patients with SARS-CoV-2 infection [47,48]. The mortality was lower in patients with compensated forms versus decompensated forms, 14% vs. 40.8% (n = 19/134 and n = 38/93) [49].

Invasive ventilation was necessary in 24% of the cases with decompensated cirrhosis, compared with 19% in patients with compensated forms [50].

One study from northern Europe found no clear link between COVID-19 and the outcome or evolution of cirrhosis [51]. A meta-analysis that focused on the outcome of COVID-19 infection in cirrhotic and non-cirrhotic patients included 40 studies with more than 900,000 participants. This showed that the COVID-19-cirrhosis association had an increased risk for severity (OR = 2.44; 95% CI, 1.89–3.16) and death (OR = 2.35; 95% CI, 1.85–3.00) [52].

The symptomatology of COVID-19 infection in cirrhotic patients has a specific course. First, acute decompensation is found in 46% of the patients, with formation or worsening of ascites and/or the onset of hepatic encephalopathy. In total, 20–58% of patients can present these alterations without respiratory symptoms connected to COVID-19 [44].

Gastrointestinal manifestations are another possible presentation for these patients, and they are thought to be connected to increased gut permeability, electrolytic changes, and systemic inflammation [49].

A series of severity scoring classifications can be used to assess the cirrhotic patients’ status, showing poorer prognosis for patients with cirrhosis and COVID-19 compared with non-COVID-19 patients [53,54].

A study from India found increased mortality for patients with acute-on-chronic liver failure (ACLF) [55]. The mortality rate of patients with ACLF–COVID-19 association was 55% [54].

ACE2 internalization by SARS-CoV-2 causes reduced ACE2 activity with further alteration in the pathway of angiotensin-II. The role of angiotensin-II in the renin-angiotensin system is essential for vasoconstriction, renal sodium retention, and promoting hepatic fibrogenesis. The reduced number of ACE2 in cirrhotic patients with COVID-19 aggravates hepatic fibrosis and portal hypertension, exacerbating the severity of the disease [56,57,58].

The interaction between SARS-CoV-2 and the liver appears to be a vicious circle, ultimately leading to multiple system organ failure (MSOF) [59]. Another study showed that COVID-19 was found to increase by 5-fold the chance of death among cirrhotic patients, respectively accounting for a 2.2-fold increase in the death risk of those with decompensated cirrhosis [46,60], but the main cause of death in cirrhotic patients infected with COVID-19 was found to be respiratory failure (71% of the cases in the study), while only 19% of the patients died of complications related to the liver [61].

## 4. COVID-19 and Cholangitis

Secondary sclerosing cholangitis is a progressive disease defined by intense fibrosis and destruction of the biliary tract, which can lead to biliary cirrhosis. Secondary sclerosis cholangitis in critically ill patients (SSC-CIP) is a form of secondary cholangiopathy that can cause a rapid deterioration of the patients [61,62].

Cholestasis in critically ill patients is mostly intrahepatic and is a result of the complex pathology behind it, such as systemic inflammatory response syndrome (SIRS), ischemic hepatitis, drug-induced liver injury, parenteral support, and ventilatory support [63]. A cholestatic pattern is less frequent in acute COVID-19 infection [61].

Secondary sclerosis cholangitis in critically ill patients (SSC-CIP) caused by SARS-CoV-2 was defined as a cholestatic liver injury that develops post-COVID-19 infection [64].

Despite emerging reports of secondary sclerosing cholangitis (SSC) in critically ill SARS-CoV-2-infected patients, there are no specific imagistic features that could be associated with the evolution of delayed progressive cholestatic liver injury, leading to cirrhosis [64].

Anatomically, the biliary system receives blood only from the peribiliary vascular plexus, while the liver parenchyma has two major blood supplies. The hypothesis of ischemia has been used to explain that severe hypotension with decreased mean arterial pressure (MAP) and longer time spent in the prone position are the triggers of SSC-CIP [65]. The incidence of SSC-CIP is directly proportional to ICU stay, even in patients without any history of biliary or liver disease. SSC-CIP is defined as a significant complication of ICU stay [66].

This new variant of sclerosis cholangitis provoked by COVID-19 is characterized by liver enzyme elevation even in patients who have not been to the ICU [67]. Pathologists have reported that severe cholangitic injury and intrahepatic microangiopathy are found in COVID-19 infection [67]. When comparing the level of RNA between airway cells and hepatic cells, there is a similar range but a lower median viral RNA in hepatic cells [68].

The binding of SARS-CoV-2 to the ACE2-R from cholangiocytes affects the barrier and the mechanism of transportation of the bile acid by affecting gene regulation. This leads to cholestasis and liver damage [69,70].

A retrospective study conducted in one center in Zurich on 34 patients admitted with COVID-19 to the ICU showed that 14 (41%) had mild cholestasis and 9 (27%) presented with a severe form of cholestasis [63]. Moreover, the ICU stay was prolonged for the last group of patients, and 4 of them developed secondary sclerosing cholangitis [63].

One study showed the case of a 38-year-old male with COVID-19 pneumonia that developed, afterward, hyperbilirubinemia, jaundice, and elevated transaminases [71]. He was hospitalized multiple times for pain, nausea, vomiting, and elevated liver enzymes, which resolved with supportive treatment. The patient underwent magnetic resonance cholangiopancreatography (MRCP), which revealed diffuse intrahepatic biliary distention and irregularity in the extrahepatic common bile duct. After performing the biopsy (cholangiocyte injury, bile ductular proliferation, canalicular cholestasis, fibrosis), the diagnosis of secondary sclerosing cholangitis was taken into consideration [71].

Biliary damage in COVID-19 patients should be taken into consideration when clinical and biological findings reveal jaundice, elevated transaminases, and/or cholestasis. Biliary imaging should be performed to confirm/infirm SSC [71].

One study that enrolled 496 patients showed that 15.4% of patients developed SSC, and patients with both chronic liver disease and COVID-19 pneumonia had a higher risk [72].

The multisystemic effect of SARS-CoV-2 has been intensively studied. The connection between COVID-19 and SSC was underlined, as several case reports have been published [73]. A middle-aged man came to the emergency room after being infected with SARS-CoV-2, with aggravating jaundice, abdominal pain, elevated liver enzymes, and dark-colored urine [73]. He underwent endoscopic retrograde cholangiopancreatography (ERCP) and MRCP, and the diagnosis of SSC was established based on the findings; he was registered for liver transplantation [73].

Another study presented chronic cholestasis and liver injury developed after COVID-19 [74]. Histopathologically, cholangiocyte injury with microvascular changes was observed. In one case, fibrosis and the presence of cytokeratin 7 metaplasia of periportal hepatocytes (an important factor for obstructive cholestasis) were reported [74].

A study presented the case of a patient who continued to have chronic cholestasis and liver injury after severe sepsis connected to COVID-19 [75]. The diagnosis of SSC was established after a liver biopsy, and ursodiol 250 mg three times a day was administered. After 6 months, the patient was discharged, and jaundice and elevated liver enzymes were ameliorated, but still persistently elevated [75].

One study published this year compared 24 patients with SSC after COVID-19 with 77 patients with SSC-CIP [66]. The median for SSC development was 91 days after COVID-19. There were no differences in most of the symptoms and transplant-free survival. Ursodeoxycholic acid treatment and albumin were administered [66]. Even though there are similarities between these two entities, the authors concluded that the course of the disease and the risk factors are unrelated between the two pathologies, and ursodeoxycholic acid administration needs further validation [66].

There is no established protocol for SSC treatment, and the last curative option is liver transplantation [76,77].

## 5. COVID-19 and NAFLD (Non-Alcoholic Fatty Liver Disease)

Non-alcoholic fatty liver disease (NAFLD) is defined as hepatic steatosis with or without inflammation and/or fibrosis [78]. The disease is further divided into NAFL—non-alcoholic fatty liver (NAFL) and non-alcoholic steatohepatitis (NASH), the main difference between them being the presence of inflammation [78]. Liver biopsy is the gold standard method of diagnosing NAFLD (staging from milder forms (steatosis) to severe forms (NASH, advanced fibrosis, cirrhosis)). Histologically, NAFLD has been defined as the presence of hepatic steatosis, ballooning, and lobular inflammation with or without fibrosis [79].

NAFLD is a heterogenous condition, mainly related to the types of lipids that accumulate, their toxicity to the liver, and to the ability of the individual to defend against it. The response of wound healing of individuals determines their capacity to recover from NASH or to develop scarring, cirrhosis, and even hepatocellular carcinoma [79].

NAFLD is becoming a leading cause of chronic liver disease worldwide because of the high incidence of both obesity and metabolic syndrome [80].

While the prevalence of the disease varies with age, sex, and demographics, it is more common in Hispano-American men aged between 40 and 50 years [78].

A systematic review revealed that COVID-19 infection has a more severe course in NAFLD patients, being correlated to ICU stay [81]. Furthermore, NAFLD is a significant risk factor for COVID-19, a finding that has been further proven accurate after the adjustment was made for obesity as a confounding factor [82]. Controversially, a few articles have stated that NAFLD does not influence the rate of mortality [83,84] or the outcome of COVID-19 [83,85]. NAFLD can be a predictor of consecutive hepatic injuries [85]. Although NAFLD might not be a major risk factor for a worse outcome in COVID-19, those patients often have other associated pathologies that may influence the course of the disease (e.g., metabolic syndrome) [61].

A case-control study in 2021 evaluated 71 patients divided into two groups: with or without fatty liver on CT scans. In total, 22 (31%) had NAFLD [86]. Severe forms of COVID-19 developed in NAFLD patients (*p* < 0.005), providing further evidence for NAFLD as a risk factor for a poor prognosis [86].

Another study confirmed that the risk for hospitalization and severity is higher in patients with NAFLD/NASH regardless of gender or race [87]. Moreover, after receiving treatment for NAFLD/NASH, obese patients have a diminished risk for hospitalization [87].

Patients with NAFLD (particularly those with NASH) often have one or more components of metabolic syndrome, to which NAFLD is linked [88]. SARS-CoV-2 infection is known to have a worse outcome in patients with high BMI or diabetes [89]. The pathophysiological mechanism is related to obesity-related poor immune responses to infection. It has been postulated that there is chronic inflammation in obese patients with pro-inflammatory citokine release mediated by the NLRP3 inflammasome [89].

NAFLD is not only associated with COVID-19, but it can increase the risk of developing all types of infections through systemic alterations, such as hyperglycemia, insulin resistance, alteration of innate immunity, obesity, and vitamin D deficiency [90].

The lockdown caused changes in lifestyle: more coffee and tobacco use and less physical activity [91]. Moreover, during the pandemic era, NAFLD and insulin resistance prevalence worsened as a consequence of the lack of screening and regular monitoring of patients [91].

Research related to fatty liver index was published evaluating 3122 COVID-19 patients from the South Korea database [92]. The aim was to correlate the fatty liver index (FLI) of NAFLD patients with the severity of COVID-19, showing that NAFLD patients have a poorer prognosis and a higher likelihood of needing mechanical ventilation, ICU admission, and high flow oxygen therapy. As a result, FLI can be used to assess the prognosis of COVID-19 [92].

SARS-CoV-2 infection increases the likelihood of developing new types of liver dysfunctions [88]. The majority of the macrophages are hosted in the liver, and as the macrophages have an impeded clearance in the SARS-CoV-2 infection, it leads to further alteration of liver function through augmented cytokine production [88]. In NAFLD patients, the polarization status of macrophages might be affected, influencing the inflammatory response to SARS-CoV-2. A study conducted in central London concluded that there was no statistically significant difference regarding mortality or ICU admissions between patients with NAFLD and those without. The main difference was that patients with NAFLD were younger and had a higher inflammatory response at the time of admission [83].

Excessive weight and NAFLD associate a pro-inflammatory status with high levels of pro-inflammatory cytokines [93] (NLR family pyrin domain containing 3—NLPR3, IL-1 [89]), increasing susceptibility to severe infection. Obese patients with NASH have a higher risk of being infected with COVID-19, as they have higher liver mRNA expression of ACE2 and TMMPRS 2 [94].

NAFLD is frequently present in patients with metabolic syndrome (hyperglycemic syndrome, hypertriglyceridemia, hypertension, raised high-density lipoprotein cholesterol HDL-cholesterol, obesity), and the influence of ACE2-R has been studied in patients with NAFLD and in those with type 2 diabetes [94]. In T2D, it was observed that there are lower amounts of ACE2-R in comparison to TMPRSS2, which showed no statistically significant difference. Furthermore, ACE2-R is more present in men with T2D than in women. We can theorize, based on the available data, that the main mechanism of the entrance of SARS-CoV-2 is not majorly altered in the livers of obese men with T2D, but there may be a lower susceptibility for liver injury in women [94].

COVID-19 enters the cholangiocytes through ACE2-R and causes direct damage with liver enzyme alteration, including albumin, GGT, ALT, and AST [95,96,97]. Moreover, the cytokine storm activated in COVID-19 affects the liver directly. TNF-*α*, a pro-inflammatory protein, is produced by adipose tissue and liver macrophages and modifies the immune response in patients with NAFLD. This process has consequences for M1 macrophages, which are suppressed, while M2 macrophages have an intense activation [95,96,97]. Taking this statement into account, it is necessary to identify a targeted treatment to reduce FLI [95,96,97].

A recent study attempted to find a connection between COVID-19 chronic sequelae and the progression to NAFLD [98]. The hypothesis was that systemic inflammation, all metabolic changes, including changes in the gut microbiome, and stimulation of liver fibrosis can be caused by COVID-19 [98]. A trend for liver enzyme alteration, steatosis, hyperplasia of Kupffer cells, and hepatobiliary congestion was revealed. The liver was indirectly affected, as the main targets were the ACE2-R [98]. The release of pro-inflammatory cytokines might trigger NASH development through their systemic effect [98]. Changes in the gut microbiome have been observed in a large number of patients with COVID-19 [98,99], but further studies are needed.

## 6. COVID-19 and Hepatitis B and C Infection

Hepatitis represents the inflammation of the liver caused by a variety of factors, frequently hepatic viruses (A, B, C, D, E). Diagnosis can be made by testing the presence of specific antiviral antigens or antibodies. Hepatitis B is caused by an enveloped virus containing an incomplete double-stranded, circular DNA genome [100]. It has different clinical outcomes depending on the patient’s status (age, immune status, or stage of the disease) [100].

Another infection is hepatitis C, which nowadays is the major cause of cirrhosis and hepatocarcinoma. It became curable with treatment development, which can lead to eradication in 98% of infected patients [101,102].

Moreover, because viral hepatic infections are a public health issue, the WHO is attempting to eradicate them by 2030 through an intense surveillance program of infected patients, accurate treatment, or vaccination [103,104].

A clinical study suggests that either current or past infection with HBV does not influence the risk of severe liver injury in SARS-CoV-2 coinfection [105].

In a large study that included SARS-CoV-2 infected patients (675 with HBV infection and 18,485 patients without signs of infection), it was observed that HBV-positive patients were older and more frail than HBV-negative ones. The COVID-19 mortality rate was evaluated at 8.2% in the HBV-negative group and 13.5% in the HBV-positive group. Moreover, COVID-19 and HBV-positive patients required intensive care unit admission and had a higher risk of MSOF [106]. In this study, it was concluded that COVID-19 and HBV infection influence the survival rate of the patients [106]. Using antiviral therapies might provide a higher risk of progression to liver failure but might reduce the risk of respiratory insufficiency [106].

Treatment for COVID-19 implies one agent that works against the SARS-CoV-2 virus itself or one against the inflammatory response of the host [105].

Liver injury in COVID-19 patients can be attributed to direct viral effects on the liver and/or hepatotoxic drugs, as proven by the elevated liver enzymes found in these patients [107,108]. The viral HBV reactivation after monoclonal antibodies (tocilizumab) and corticosteroid usage raises the need for careful screening before prescription [107,108].

The prognosis of infection with SARS-CoV-2 in HBV patients depends on the stage of liver disease, as proposed by Shanshan Yang et al. [109]. Therefore, HBV infection with HBeAg (+) can cause a bad outcome for patients compared to infection with HBeAg (−) [109]. Moreover, age is essential for the prognosis of HBeAg (+) VHB infection and for HBV reactivation [109]. Moreover, a great number of these patients presented with abnormal liver function and severe status requiring ICU admission [109].

Chen et al. observed that there are no differences in hospital length of stay between HBV-positive and HBV-negative coinfected patients [110]. He et al. observed the same aspect and also showed that coinfection is not associated with a poorer prognosis [111].

On the other hand, another study observed that patients who have a hepatic infection during the coinfection stage have a higher liver alteration. A multicentric study from Wuhan, China, observed that abnormal AST or DBIL levels are not risk factors for COVID-19 mortality, but they can be considered independent risk factors [112].

A study published in the *American Journal of Gastroenterology* reported that HBV incidence among patients with SARS-CoV-2 infection was lower compared with the overall incidence of HBV infection in the Chinese population [113]. It has been theorized that the excess of T lymphocytes can affect the ability of these patients to respond to other viruses so that the cytokine storm is reduced and the severity of COVID-19 is decreased [113].

During the pandemic, the number of hepatitis C virus RNA tests decreased by 62% in March 2020 and remained at 39%, below the baseline, by July 2020 [114]. When it comes to hepatitis C and COVID-19, the use of immunosuppressants is associated with a risk of HCV reactivation [115,116].

The two pathophysiological mechanisms involved are the stimulation of viral replication, directly, and the suppression of the immune system [117]. These two mechanisms might cause HCV reactivation [117]. Moreover, HCV viremia increases during corticosteroid treatment and can return to the baseline when the medication is discontinued [117].

Having a history of HCV infection might make a patient more susceptible to severe respiratory complications, without the influence of any other comorbidity, a biological marker at admission, or COVID-19 liver injury [118]. The effect seems to be correlated to the extrahepatic effects of HCV, stimulating ACE-2/TMPRSS mechanisms, the inflammatory process, and endothelial dysfunction [119].

A multicenter retrospective study evaluated 125 patients with chronic hepatitis and COVID-19 (64 patients with cirrhosis). They presented with a large variety of symptoms, but cough, core throat, fatigue, myalgia, and diarrhea were more frequent in cirrhotic patients. They discovered many independent factors that can influence the outcome of patients: male gender, diabetes mellitus, and cirrhosis [118].

When the HCV and COVID-19 coinfection was evaluated for the inflammatory response and the cytokine types involved, the levels of interleukin 17 and 6 were found to be higher in coinfected patients compared with patients infected only with SARS-CoV-2 [120].

Sofosbuvir, tenofovir, and ribavirin, an antiviral treatment used for HBV and HCV, have been shown to have activity against infected patients with SARS-CoV-2 through their binding to the RNA-dependent-RNA polymerase (RdRp) of the coronavirus [121], but future trials are needed.

Furthermore, a form of transient hepatitis in a patient that tested positive for the SARS-CoV-2 virus, named COVID-19-induced hepatitis (CIH), was described. It might progress with elevated ALT and AST, non-obstructive jaundice, and inflammatory infiltration of the liver parenchyma [122]. An increased AST/ALT ratio is linked to systemic inflammation, while a low ratio is related to liver injury [123].

With ongoing research about COVID-19 and its connection with liver alteration going on, an isoquinoline alkaloid, called berberine, was found to have important activity against COVID-19, with multiple effects—antiviral, anti-allergy, and anti-inflammatory—ensuring hepatoprotection against drugs and infection-induced liver injury while reducing oxidative stress [124]. Berberine can have an antiviral effect against various agents (influenza, hepatitis B, hepatitis C, cytomegalovirus, alphavirus, and papillomavirus) [124]. The mechanism of this agent is still under evaluation, but it is clear that it can increase IFN-γ levels by intense stimulation of CD8 T cells and can inhibit histamine release from mast cells. Moreover, ACE2, IL-1α, IL-8, IL-6, and chemokine ligand 2 (CCL-2) are inhibited by berberine administration. It can be beneficial not only for COVID-19 treatment but also for viral infection treatment or eradication [124].

One study proved that berberine has a beneficial role in treating the bacterial infections associated with COVID-19 and in reducing hydroxychloroquine toxicity [125].

Berberine can be a promising treatment for hepatic C infection. It has been found to affect the entry/fusion into the cell of the virus. Moreover, it inhibits viral pseudoparticles E1/E2 [126,127]. It might become a possible antiviral treatment for HBV infection because of its effect on mitogen-activated protein kinase (MAPK) pathways. Kim et al. suggested that berberine acts like a p38 MAPK inhibitor and, in this way, can cause HBsAg suppression [127,128].

Another promising molecule is 8-hydroxydihydrosanguinarine, a phyto-alkaloid that interferes in the connection of S protein to ACE2, which indicates a potential therapy for COVID-19 [129], but further studies are needed.

## 7. Conclusions

The systemic inflammation developed during COVID-19 may change the course of hepatic preexisting diseases, with additional morphofunctional alterations of the liver, and may cause a persistent effect from that point forward.

The impact of COVID-19 infection on cirrhotic patients is significant, influencing their prognosis and outcome. Medical societies have raised awareness about the exposure of these patients.

There are premises of a new emerging entity caused by the SARS-CoV-2 virus—the secondary sclerosis cholangitis in critically ill patients, without any preexisting liver conditions.

The polymerization used to treat SARS-CoV-2 infection is continuously changing, exposing the patients with preexisting liver alterations to more and more possible complications.

The effects of COVID-19 on the liver are a challenge for doctors, raising concern about the medical approach and treatment of patients with preexisting hepatic conditions.

## Data Availability

Not applicable.
